# Supplementary material for: Overexpression of Murine Rnaset2 in a Colon Syngeneic Mouse Carcinoma Model Leads to Rebalance of Intra-Tumor M1/M2 Macrophage Ratio, Activation of T Cells, Delayed Tumor Growth, and Rejection
Source: Cancers (Basel). 2020 Mar 18;12(3):717. doi: 10.3390/cancers12030717 (PMC7140044; doi:10.3390/cancers12030717)
Supplement: Supplementary file 1 [file cancers-12-00717-s001.pdf]

Supplementary Materials: Overexpression of Murine *Rnaset2* in a Colon Syngeneic Mouse Carcinoma Model Leads to Rebalance of Intra-Tumor M1/M2 Macrophage Ratio, Activation of T Cells, Delayed Tumor Growth, and Rejection

Annarosaria De Vito, Paola Orecchia, Enrica Balza, Daniele Reverberi, Debora Scaldaferri, Roberto Taramelli, Douglas M. Noonan, Francesco Acquati, and Lorenzo Mortara

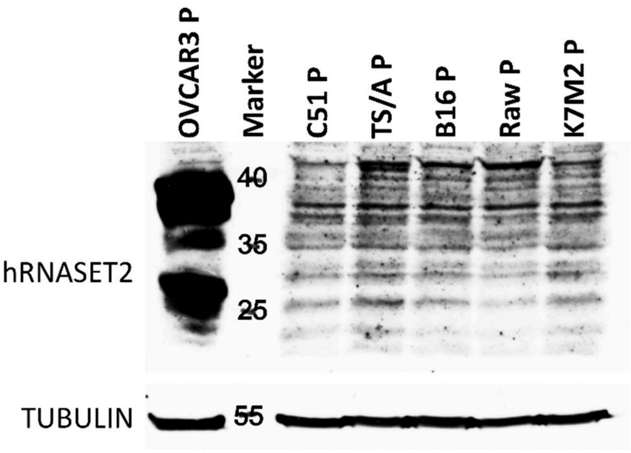

Figure S1. Immunoblot analysis of Rnaset2 in five parental murine tumor cell lines (including C51 and TS/A) using a polyclonal anti-human RNASET2 antibody which cross-reacts with the mouse protein [1]. The human OVCAR3 ovarian cancer-derived cell line, which expresses high endogenous RNASET2 protein, was included as a positive control. All murine cell lines tested are considered Rnaset2 negative, since all bands detected in the murine protein samples by the human anti-RNASET2 antibody represent non-specific products.

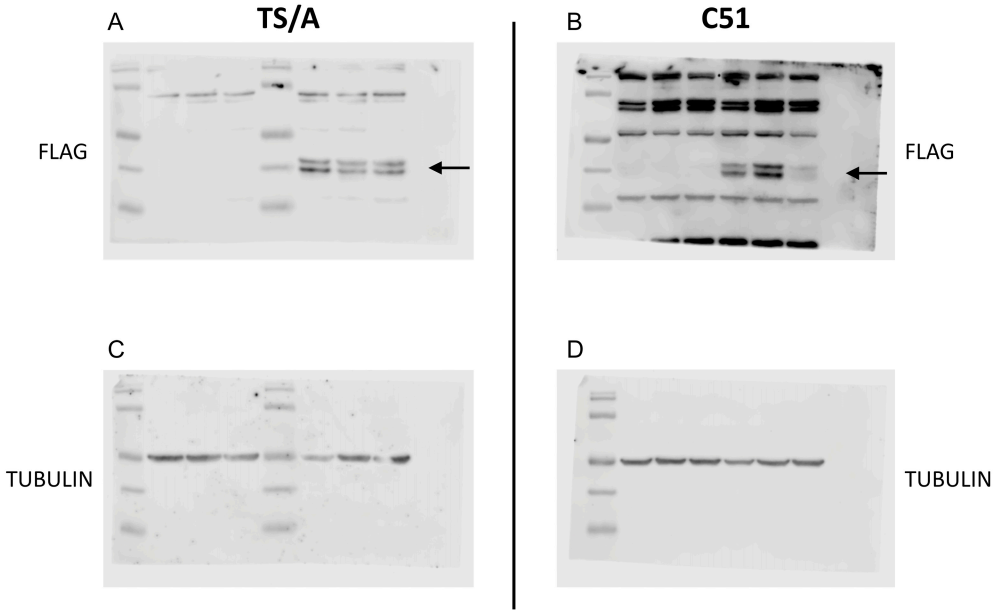

Figure S2. Whole-filter images of the immunoblot analysis for Rnaset2-FLAG expression in TS/A and C51 murine cell lines. Panel **A** and **B**: an intracellular lysate was analyzed for Rnaset2-FLAG expression in TS/A and C51 E and FL Rnaset2 clones, respectively. Panel **C** and **D**: the same filters were probed with anti-tubulin polyclonal antibody for normalization.

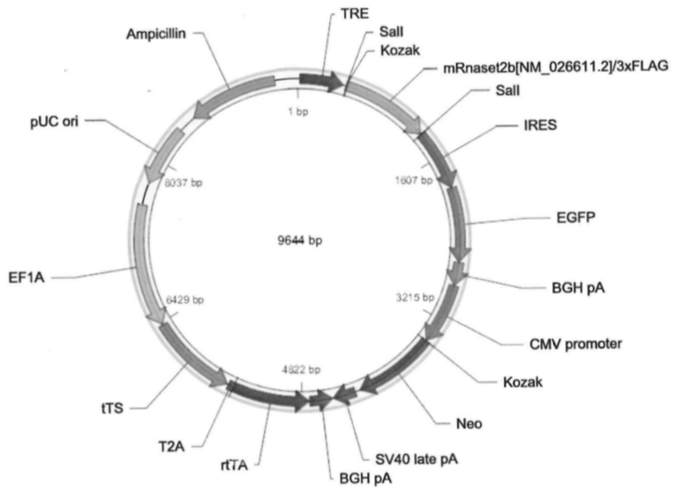

Figure S3. Schematic representation of the inducible vector from VectorBuilder Company.

Reference

1. Campomenosi, P.; Salis, S.; Lindqvist, C.; Mariani, D.; Nordström, T.; Acquati, F.; Taramelli, R. Characterization of RNASET2, the first human member of the Rh/T2/S family of glycoproteins. *Arch. Biochem. Biophys.* **2006**, *449*, 17–26.

© 2020 by the authors. Licensee MDPI, Basel, Switzerland. This article is an open access article distributed under the terms and conditions of the Creative Commons Attribution (CC BY) license (http://creativecommons.org/licenses/by/4.0/).
